# Growth of Carbon Nanotubes Using Mixed-Metal Catalysts That Include Heavy Refractory Metals as Catalyst Stabilizers

**DOI:** 10.1021/acsomega.5c03084

**Published:** 2025-09-02

**Authors:** Wonjung Park, Matthew G. Boebinger, Liam Collins, Alexander A. Puretzky, Ilia N. Ivanov, David B. Geohegan, Michael J. Bronikowski

**Affiliations:** 1 Dept. of Chemistry and Biochemistry, University of Tampa, Tampa, Florida 33606, United States; 2 Center for Nanophase Materials Science, 6146 Oak Ridge National Laboratory, Oak Ridge, Tennessee 37830, United States

## Abstract

We report new findings from studies of a novel technique to augment the growth of CNTs. In this method, the catalytic metals used for CNT growth are combined with high-melting-point heavy refractory metals, which serve to stabilize the metal catalyst nanoparticles, from which the CNTs nucleate and grow. Our study investigates the combination of an iron catalyst with tungsten and osmium refractory stabilizers. We find that both tungsten and osmium enhance the lifetime of the iron catalyst particles by a factor of approximately two. Tungsten stabilizer resulted in production of CNTs longer by a factor of 1.25 compared to those produced using a pure iron catalyst, while the osmium stabilizer did not significantly affect the ultimate length of the CNTs due to the decreased CNT growth rate with a combined Fe/Os catalyst. These results suggest a possible new route toward producing CNTs with lengths sufficient for materials applications.

## Graphical abstract

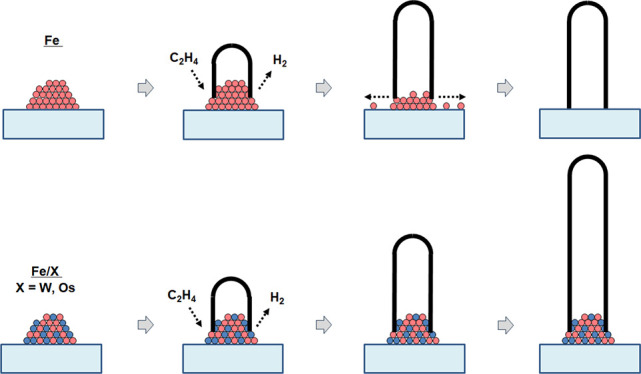

## Introduction

Since the discovery of carbon nanotubes (CNTs)^1^ and the demonstration of their production in large-scale quantities,^2^ there has been growing interest in integration of CNTs in many applications to capitalize on their exceptional mechanical and electrical properties.^3^ CNTs are normally grown in bulk by using catalytic chemical vapor deposition (CVD). Carbon-containing gases like methane, ethylene, or acetylene are passed over nanometer-sized particles of catalytic metals including iron or cobalt. At elevated temperatures, these gaseous precursors decompose upon the catalyst particle to give carbon, which precipitates in the form of a nanotube whose diameter correlates with the particle’s diameter. This nanotube grows away from the particle upon which it nucleated, with new carbon continually added at the nanotube/particle interface by the ongoing decomposition of the carbonaceous gases. One of the most common methods to grow CNTs in this way involves flowing carbonaceous gases over fixed catalyst nanoparticles supported on a substrate held at elevated temperature, which results in a dense mat or forest of CNTs that grow aligned parallel to each other as they grow perpendicularly away from the substrate.

To fully leverage the unique properties of CNTs in applications such as cables, wires, and ultrastrong composites, there is a necessity for large-scale production of carbon nanotubes with lengths on the order of tens of centimeters to meters or more^3^. Thus, much work on the bulk production of CNTs has focused on maximizing the ultimate length to which CNTs could be grown. Initial investigations of bulk CNT growth, made shortly after their discovery, typically gave CNTs with lengths of only a few hundred microns.^4,5^ The field witnessed significant progress following the discovery that the inclusion of water or other oxygen-containing molecules with the carbon feedstock gas could enhance CNT growth.^6^ However, until recently, the maximum length to which CNTs could be grown remained at approximately 2 cm.^7^ Very recently, Sugime et al.^8^ demonstrated that inclusion of trace amounts of organometallic compounds containing iron and other metals could increase CNT growth beyond this length: this group produced dense CNT mats with lengths up to 14 cm. The quest for further advancements continues with ongoing interest in identifying methods to augment the maximum growth length of CNTs.

The mechanisms of CNT nucleation, growth, and growth cessation have been extensively studied.^6–47^ CNT growth occurs when carbon-containing molecules decompose upon the surface of nanometer-scale particles of catalytic metals (e.g., iron) to form elemental carbon on the particle’s surface. If the particle is in the correct size range, typically 1–10 nm, the carbon will form into a carbon nanotube, which grows away from the nanoparticle as additional carbon is added at the nanotube/particle interface in an ongoing way. Many studies have shown that CNT length is limited because, under typical CVD conditions, CNTs nucleate and grow for only a limited time before growth ceases. Among the principal causes of this growth cessation is instability of the catalyst particles from which the CNTs nucleate and grow; these particles can change and deactivate over time via several mechanisms including Ostwald ripening, particle coalescence, catalyst poisoning, and diffusion of the catalyst into the substrate material.^9–25^ Thus, much recent work has focused on methods to increase the lifetime and overall stability of the catalytic particles.^7,26–35^

One methodology used to stabilize catalyst particles for CNT growth consists of combining the catalyst metals, typically fourth-row transition metals such as iron or cobalt, with heavy refractory metals, typically fifth or sixth row transition metals with substantially higher melting points. Li and co-workers have used this method to reliably produce single-walled CNTs (SWCNTs) with specified, constant chiralities.^34,35^ By combining the cobalt catalyst with tungsten stabilizer, this group was able to grow SWCNTs with high abundances of specific chiralities, as opposed to the wide distribution of chiralities yielded by catalysts of pure cobalt. Using a combination of high-resolution transmission electron microscopy (HRTEM), environmental TEM (ETEM), and other techniques, Li and co-workers showed that this constancy of SWCNT chiralities arose due to the stability of the intermetallic Co_7_W_6_ nanocrystal particles formed with this combination of metals, even up to 1100 °C: these particles did not undergo the morphology and size-distribution evolution seen in catalysts of pure Co. While this group did not investigate the ultimate length achieved by CNTs grown from pure Co versus Co/W catalysts, they did observe that, under CVD growth conditions, the Co/W particles were much more stable and long-lived than particles of pure Co. Co/W nanoparticles maintained their initial morphology and structure at 1000 °C in an atmosphere of CH_4_ for up to 487 s (the longest time reported), whereas Co nanoparticles were morphologically unstable when heated in CH_4_ and underwent transformation into CoC_3_, melting and sublimation in as little as 36 s at a much lower temperature of 700 °C.^34^ Similar effects have been observed by Amama and co-workers,^36^ who used ruthenium as a stabilizer with cobalt catalyst to grow SWCNTs with narrow, reproducible distributions of chiralities.

Our group has used a combination of catalyst metals with refractory stabilizers to produce enhanced-stability catalyst particles, which increased both CNT-growth lifetimes and ultimate CNT lengths^37^ when CO was used as carbon source gas. For example, addition of rhenium stabilizer to molybdenum catalyst particles resulted in an increase by a factor of approximately four of both CNT growth lifetimes (6 h vs 1.5 h) and ultimate CNT lengths (250 μm vs 60 μm) at a growth temperature of 1100 °C.^37^ In that work, these results were interpreted as the refractory metal stabilizing the catalyst particle by acting as a “diffusion inhibitor” (or “Ostwald ripening inhibitor”): the refractory binds the catalyst atoms strongly enough to prevent their detachment from one catalyst particle and diffusion across the substrate surface to bind to another potentially larger particle with a higher per-atom binding energy. In this way, the refractory prevents the ongoing Ostwald ripening that ultimately results in catalyst particle deactivation: it slows the particles’ deactivation through growing too small or too large to support CNT growth, stabilizing them and leading to longer catalyst particle lifetimes and longer ultimate CNT lengths.

Here, we show that, when ethylene (C_2_H_4_) is used as the carbon source gas, inclusion of the heavy refractory metals tungsten (MP: 3422 °C) or osmium (MP: 3033 °C) with iron catalyst (MP: 1538 °C) increases the lifetime of the catalyst particles by a factor of approximately two. C_2_H_4_ and similar hydrocarbons are commonly used to grow CNTs and can give much faster growth rates (up to 100 μm/min^8^) than the slower CO disproportionation reaction used in our group previously. Thus, the Fe/W and Fe/Os catalyst systems constitute the first demonstration of our diffusion inhibitor concept using CNT growth chemistry of the type that will ultimately be needed for scale-up to the large-scale production of CNT materials. Note that the primary goal of this work has been to demonstrate increased catalyst lifetime and ultimate CNT length using diffusion inhibitors with C_2_H_4_ feedstock under a given set of CNT growth conditions, which are held constant for both pure Fe and for Fe-plus-inhibitor catalyst systems. Note also that preliminary reports of some of these results have been published previously.^38,39^ In the current work, we expand on previous results and carry out a detailed analysis of the time evolution during growth of both CNTs and catalyst particles, using a combination of SEM, TEM, micro-Raman Spectroscopy, and AFM. We find that inclusion of tungsten results in the growth of CNTs longer by a factor of 1.25 than those produced using a pure iron catalyst, while osmium inclusion does not significantly affect the ultimate CNT length. Our results show that under the given set of experimental conditions used in our studies, CNTs can grow for longer times and to longer lengths because of refractory-metal stabilization of the catalyst particles. These results are consistent with the previous interpretation that the refractories inhibit Ostwald ripening and other particle morphological changes that would otherwise result in deactivation of the particles.

## Results

### CNT Growth: Length vs Time

In these investigations, we used CNT growth conditions and parameters (temperature, reactant gas flow rates and concentrations, etc.) that had been optimized for CNT growth from pure Fe catalysts in our previous work;^38,39^ see “Materials and Methods” section below for specifics of all growth conditions used. These conditions had been found to be optimal for growth of CNTs from pure Fe catalysts and were used for mixed-metal catalysts in order to have standard conditions for all growths, to allow direct comparison of results. To investigate the effect of catalyst stabilizers on CNT growth and ultimate CNT length, we first studied the dependence of the CNT length on growth time. CNTs grown using catalysts either of pure Fe or of Fe mixed with W or Os resulted in dense CNT mats, as described above. Figure 1 shows an example of such a CNT mat grown from Fe/W catalyst under the conditions described.

**Figure 1:**
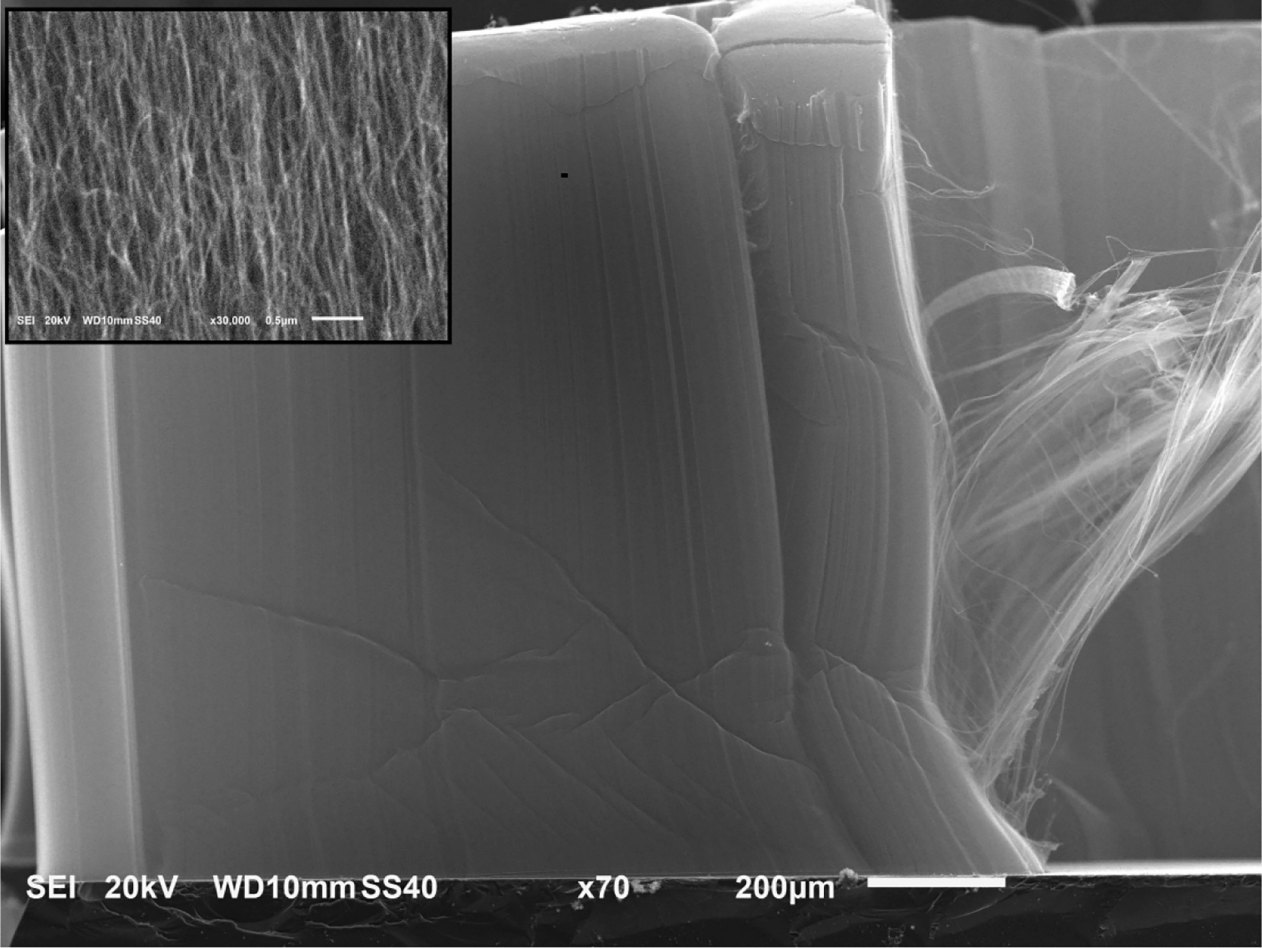
SEM image of a dense mat of CNTs grown from C_2_H_4_/H_2_ using Fe/W mixed-metal catalysts, growth time: 30 min. Inset shows fine CNT structure.

To investigate the time-dependent growth of CNT length, we conducted a series of growth runs for different catalyst combinations, with each run varying in duration between 2 and 60 min. The CNT lengths, measured using SEM, were consistent across growth runs and catalyst samples with a typical reproducibility rate within 10%. The observed CNT growth pattern aligns with previously observed behavior: initially, the nanotubes grow at an approximately constant rate. However, after a certain time, the growth rate slows and eventually stops. Significantly, metal films composed entirely of tungsten or osmium did not support any CNT growth. This suggests that the catalytic activity essential for CNT formation is uniquely attributed to the iron catalyst. Two types of metal catalyst films were used in these studies. The first consisted of mixtures of Fe with either W or Os stabilizers, always in the ratio of 50% Fe to 50% stabilizer. These catalysts effectively consisted of 5 Å (0.5 nm) thickness each of Fe and stabilizer and are referred to as Fe5W5 or Fe5Os5 in this discussion; the total thickness of the metal film for these mixed-metal samples was always 10 Å, or 1.0 nm, although the preparation procedure ensures that the atoms of catalyst and stabilizer metals are thoroughly intermixed rather than being present as separate 0.5 nm-thick layers. The second type of catalyst consisted of pure Fe catalyst with thickness of either 5 or 10 Å and are referred to in the discussions below as Fe5 and Fe10, respectively. Results for the pure Fe catalysts have been reported previously^39^ and are reproduced here with permission. These Fe catalyst films were used for comparison to the mixed-metal catalysts and represented pure-catalyst films (with no refractory stabilizer) with the same total amount of catalytic iron (5 Å) and the same total metal thickness, 10 Å. Figure 2 provides plots that delineate the relationship between the measured CNT length and growth time for the catalysts Fe10, Fe5, and Fe5W5. The data points in these plots were computed from the averages of measured CNT length over typically 2–5 CNT growth runs.

**Figure 2:**
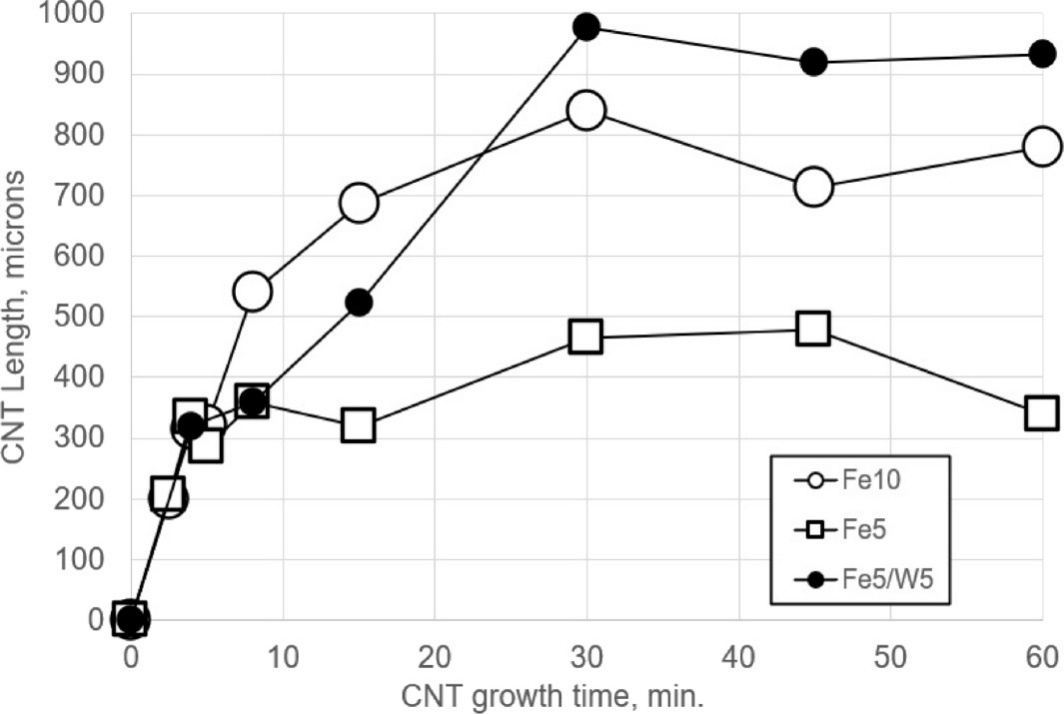
CNT length vs growth time for catalysts of pure iron and an Fe/W mixed metal. Plots for Fe10 and Fe5 are reproduced from ref 39 with permission.

In Figure 2, the growth curve for Fe5 shows rapid initial CNT growth, but only for a short time: the maximum CNT length of approximately 400 μm is reached after only 4–5 min, with no significant systematic lengthening after this length is reached, although the data of Figure 2 do show substantial run-to-run variation in the maximum CNT length reached. When using 5 Å of pure iron, CNT growth terminates quite abruptly after initiation. The growth curve for Fe10 shows a more stable situation. Here, the CNTs lengthen smoothly for approximately 15 min before slowing and stopping, again with some run-to-run variation in the final CNT length reached. For 10 Å Fe films, no systematic lengthening of the CNTs occurs after 15 min of growth time.

The behavior of CNT growth undergoes a substantial change when a refractory stabilizer is incorporated. The Fe5W5 growth curve depicted in Figure 2 demonstrates a steady elongation of CNTs grown from this mixed-metal catalyst, persisting up to 30 min of growth time, after which growth comes to a halt. When compared to the growth curve of 5 Å of iron alone, considering tungsten simply as an additive to the 5 Å of catalytic iron, the difference is quite remarkable. The Fe5W5 catalyst shows growth for 30 min, yielding CNTs with a final length of approximately 1000 μm. This is in stark contrast to the CNTs produced by 5 Å of iron alone, which only grow for around 4 min and result in CNTs merely 400 μm long. Clearly, inclusion of refractory stabilizer increases both the growth time and the ultimate CNT length by substantial factors, in this case, factors of 7 and 2.5, respectively.

Comparing the Fe5W5 catalyst to the pure Fe film of equivalent total thickness, Fe10, the differences are still present, although not as pronounced. CNTs grown from Fe10 grow for approximately 15 min and yield CNTs of length approximately 800 μm, giving the Fe5W5 catalyst growth time and ultimate length enhancement factors of about 2 and 1.25, respectively. It is clear from these results that inclusion of *W* as a refractory stabilizer with an Fe catalyst substantially increases both the lifetime of the catalyst particles and the ultimate length to which the resulting CNTs grow. From Figure 2, it is also apparent that the growth rate of CNTs grown from Fe5W5 is substantially slower than for pure Fe: CNTs from the Fe5 film grow to 400 μm in 4–5 min, for a growth rate of 80–100 μm/min (similar to growth rates reported in the literature^7,8^ ), while CNTs from Fe5W5 grow to approximately 950 μm in 30 min, or 32 μm/min. Apparently, the Fe/W combined catalyst has less catalytic activity for CNT growth than does pure Fe. Nevertheless, the increase in catalyst lifetime more than compensates for the deceased growth rate so that CNTs from Fe5W5 do achieve greater final length. Table 1 summarizes the observed initial CNT growth rates, growth lifetimes, and ultimate lengths reached by the CNTs.

**Table 1:** Initial CNT Growth Rates, Growth Times, and Ultimate CNT Lengths for CNTs Grown from Pure Fe (5 and 10 Å) and Mixed-Metal Fe/W (5Å/5Å) Catalyst Films

catalyst	initial growth rate (μm/min)	growth time (min)	ultimate length (μm)
Fe (5 Å)	84	4	390
Fe (10 Å)	67	15	780
Fe/W (5Å/5Å)	32	30	940

Use of Os as a catalyst stabilizer for Fe yielded results qualitatively similar to those observed for W, though less pronounced. See Supporting Information section S1 for complete Fe5Os5 results.

### CNT Growth: Length vs Catalyst Fractional Composition

Figure 2 shows that refractory stabilizers can prolong the lifetime of catalyst particles and in some cases result in longer CNTs. Given this result, it is of interest to inquire how large a fraction of stabilizer a mixed-metal catalyst can contain and still grow CNTs, and whether catalyst lifetime enhancement can be increased by increasing the fraction of stabilizer metal compared to the catalytic metal. For this purpose, a series of Fe/W and Fe/Os catalysts were prepared with ever increasing fraction of stabilizer (and decreasing Fe fraction), with the total metal film thickness kept constant at 10 Å for comparison with the 10 Å pure Fe catalyst. These “catalyst lean” mixed metal films had Fe thickness as low as 1 Å, with the balance made up of refractory stabilizer. For comparison, new catalysts were also prepared of pure Fe with these same thicknesses, with no stabilizer metal. CNTs were grown from all of these catalysts under conditions identical to those used above, with a growth time of 30 min. The results are shown in Figure 3.

**Figure 3:**
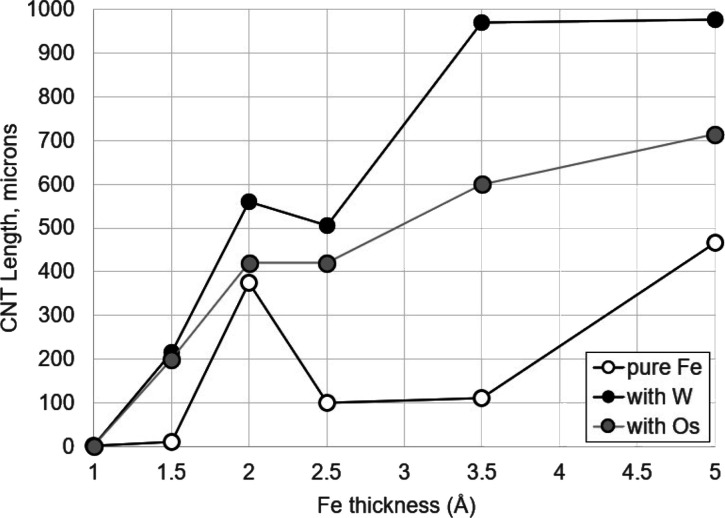
CNT length (30 min growth time) vs Fe catalyst thickness for catalysts of pure Fe and catalysts of Fe mixed with W or Os stabilizer, with stabilizer always added to give a total metal thickness of 10 Å.

Figure 3 shows that substantial CNT growth (200 μm or more) occurs using Fe/W or Fe/Os catalysts with Fe content as little as 1.5 Å (15% of total, with 85% W or Os), and that, at an Fe content of 3.5 Å (35%), CNT growth is already comparable to or greater than the maximum growth observed from films of 10 Å of pure Fe under these conditions. By contrast, films of pure Fe less than 5 Å thick give minimal CNT growth, typically 100 μm or less. Clearly, with these lower catalyst amounts, either refractory reaction results in a much greater degree of stability of the catalyst particles, much longer catalyst lifetime, and ultimately growth of much longer CNTs. Note also that extremely small amounts of Fe, 1 Å or less, give little to no CNT growth, even with stabilizer present. Interestingly, CNT growth using exactly 2.0 Å of Fe gave anomalously high growth, causing deviation of the curves from monotonically increasing CNT length with Fe thickness for both pure Fe and Fe/inhibitor combinations. This deviation was reproducible between growth runs and between samples of the catalyst-coated substrate prepared on different days. We do not currently have an explanation for this observation, but it clearly represents an anomaly in CNT growth behavior under our experimental conditions that will warrant further investigation in future studies.

The above data were collected for a CNT growth time of 30 min for all samples. Based on the observed CNT growth times for 5 and 10 Å of pure Fe (4 and 15 min, respectively), it is likely that CNTs grown from thinner films of pure Fe will reach their maximum length within 30 min. On the other hand, the higher stabilizer-to-catalyst ratio in the Fe-minority catalysts could possibly extend CNT growth times beyond the 30 min observed for Fe5W5. Investigations into growth behavior as a function of Fe content in Fe/W and Fe/Os catalysts are ongoing. Future work will also explore how the CNT growth time varies with the catalyst-to-stabilizer ratio in these mixed-metal catalysts.

### TEM Analysis

TEM imaging was carried out on CNTs grown from Fe5, Fe10, Fe5W5, and Fe5Os5 catalyst films. All samples showed predominantly double-walled CNTs (DWCNTs), with some CNTs with more walls, typically 3–6. Interestingly, no single-walled CNTs (SWCNTs) were observed in any sample. All CNTs observed had diameters in the range 5–10 nm, with no significant differences noted in the diameters or numbers of walls of CNTs grown from any of these catalyst systems. CNTs from several of the “catalyst lean” metal films that contained Fe as a minority component were also analyzed using TEM, including catalysts with as little as 15–20% Fe. These CNT mats likewise consisted primarily of DWCNTs with diameters of 5–10 nm. Figure 4 shows TEM images of CNTs grown from various catalyst systems.

**Figure 4:**
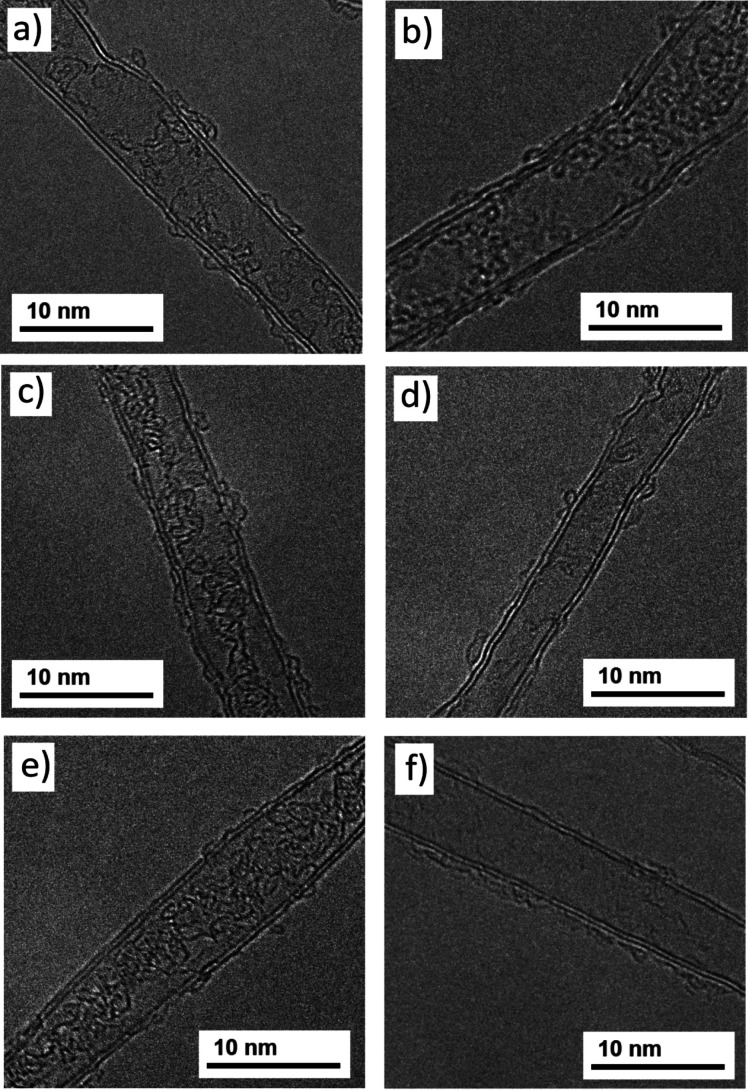
TEM images of double-walled CNTs grown using catalyst films: (a) Fe10Å, (b) Fe5Å, (c) Fe5Å/W5Å, (d) Fe5Å/Os5Å, (e) Fe2Å/W8Å, and (f) Fe2Å/Os8Å. Images in panels (a) and (b) are reproduced from ref 39 with permission.

We also observed occasional metal nanoparticles entrained inside of the CNTs in our mats. Elemental analysis by energy-dispersive X-ray scattering (EDS) spectroscopy of CNT samples grown from Fe5W5 catalyst showed that these particles consisted of iron (see Supporting Information section S2 for complete details of metal particle TEM/EDS analysis).

### CNT Alignment vs Time: Pure Fe vs Fe/W Mixed Catalyst

Previous work has shown that cessation of CNT growth in dense CNT mats is often preceded by a decrease in the degree of alignment of the CNTs in the mat.^8,19,40^ This alignment decrease arises from decreasing the CNT number density as individual CNTs cease growing. If it is correct that inclusion of refractory metal stabilizes catalyst particles and slows their degradation with time, then it might be expected that CNT alignment in the growing mat would be preserved for longer times and for greater lengths of CNT if the catalyst is combined with a stabilizer. To investigate this effect, we acquired high-magnification SEM images of the sides of the CNT mats from the Fe10 and Fe5W5 catalysts at various points along these CNTs’ lengths. Figure 5 shows SEM images of the CNT mats acquired at various distances from the top of the CNTs. The mats in these samples were grown using a growth time of 30 min, and the CNTs of these mats reached lengths typical of the two catalysts, 850 μm for Fe10 and 1250 μm Fe5W5.

**Figure 5:**
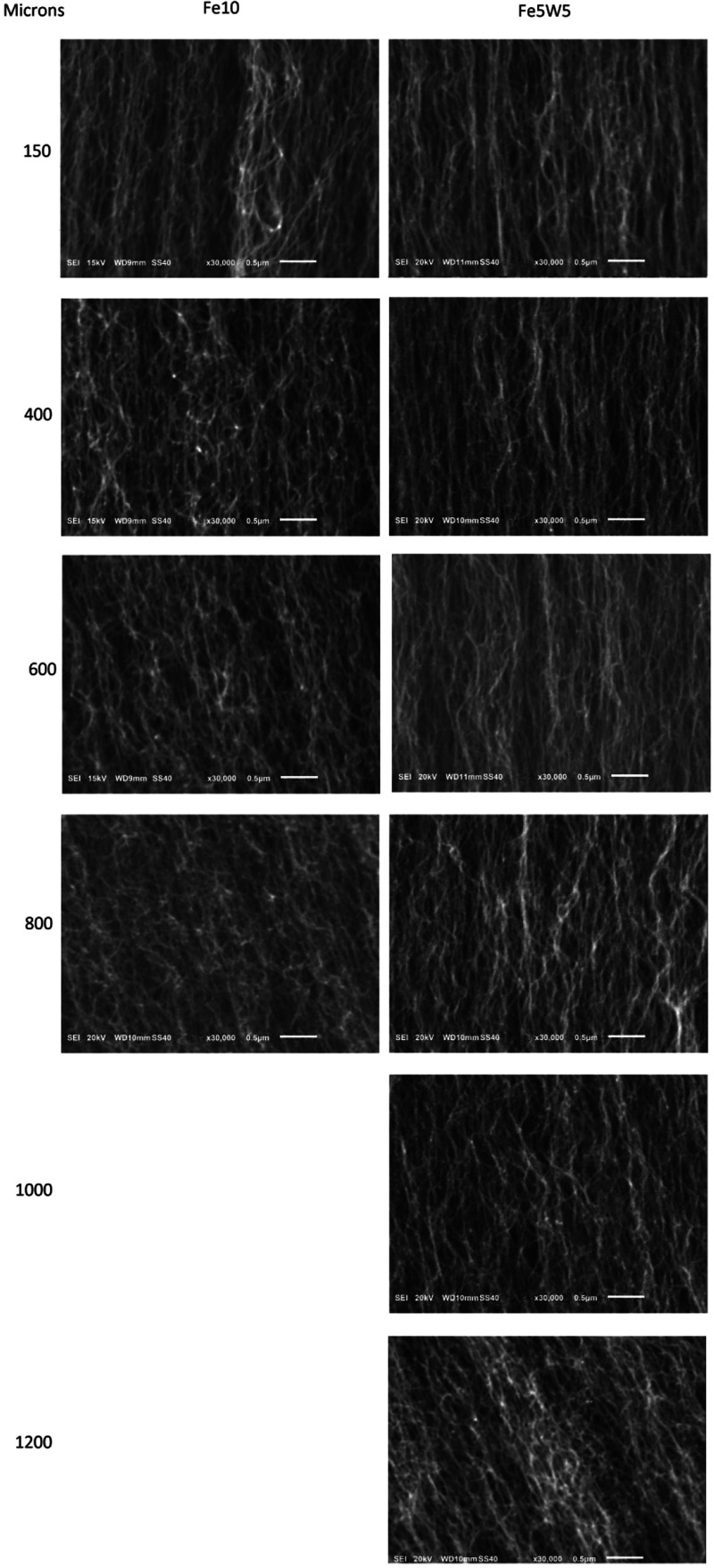
SEM images at 30,000× magnification of CNT mats grown from catalyst films of Fe10Å and of Fe5Å/W5Å, at various points along the CNTs’ lengths. The column labeled “*Microns*” indicates microns from tops of CNT mats.

Figure 5 shows both mats initially well aligned, with images taken 150 μm from the CNTs’ tops showing good alignment. By 400 μm of growth, differences are observed. The CNTs grown from Fe10 begin to show a decrease in alignment with more random tangling occurring within the mat. This misalignment persists and increases throughout the remainder of the CNT growth for this mat to its final length, 850 μm. By contrast, the CNTs grown from Fe5W5 continue to show good alignment within the growing mat up to 600 μm of growth. Only at 800 μm of growth do random tangling and misalignment begin to set in, with these persisting for the remainder of the growth, 1250 μm for this mat. These observations show that the Fe5W5 catalyst particles remain stable and catalytically active longer than particles of pure Fe. The difference is all the more notable because, as noted above, the Fe5W5 CNTs grow more slowly than those from Fe10. Referring to Figure 2, CNTs from Fe10 reach 400 μm in length after approximately 6 min; loss of alignment by this point indicates that, after 6 min under CVD conditions, a substantial fraction of the catalyst particles growing CNTs have already deactivated.

By contrast, CNTs from Fe5W5 reach 600 μm length after approximately 18 min of grown; good alignment at this point indicates that the catalyst particles have not evolved to the point of growth cessation even after 18 min. Only after approximately 24 min of growth, at 800 μm length, do we begin to see degradation of CNT alignment, with its implied catalyst particle growth cessation. The Fe/W mixed-metal particles clearly remain stable for substantially longer time than do particles of pure Fe.

### Raman Spectroscopy of CNT Mats: Pure Fe vs Fe/W Mixed Catalyst

Position-dependent Raman spectroscopy of CNT mats can reveal much about time-dependent changes in CNT morphology and, by inference, the morphology of the catalyst particles from which the CNTs are growing.^7,8,31,40–42^ To investigate these changes, Raman spectra of CNT mats grown from Fe10 and Fe5W5 were acquired as a function of distance from the CNTs’ tips by focusing the Raman laser onto the exposed sides of the CNT mats at locations of varying distance from the tops of the CNT mats. Figure 6 shows important sections of these Raman spectra, including the small-diameter single-walled CNT (SWCNT) Raman breathing-mode (RBM) region, 100–350 cm^–1^; and the carbon D and G bands near 1330 and 1570 cm^–1^, respectively. The Raman G band arises from in-plane C–C vibrations within the sp^2^-hybridized graphene layers, while the D band is associated with disorder within the CNTs. All of these spectral regions are shown in Figure 6 from Raman spectra of the sides of the CNT mats acquired at increasing distances from the top of the mats; the larger distances correspond to portions of the CNT mats that grew later in of CNT growth and hence from catalyst particles whose morphology may have changed during that growth.

**Figure 6:**
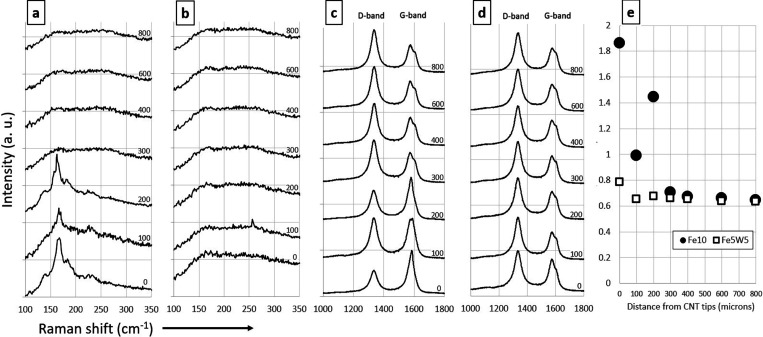
Raman spectra of CNT mats grown from Fe10 and Fe5W5, at various distances from the tops of CNT mats. Notation on spectra indicates distance in microns from the top of the mat. (a) RBM region of CNTs grown from Fe10; (b) RBM region of CNTs grown from Fe5W5; (c) carbon D and G band region of CNTs from Fe10; (d) D and G band region of CNTs from Fe5W5; (e) *I*_G_/*I*_D_ intensity ratios vs distance from tops of mats.

Figure 6 shows that CNT mats from Fe10 catalyst had substantial numbers of SWCNTs present early in the growth, based on peaks observed in the SWCNT breathing-mode region. Over time, the intensity of these peaks decreases, until, by a depth of 300 μm, no SWCNT peaks are observed. This same pattern has been observed previously for mats of long CNTs,^40,41^ and indicates that small catalyst particles needed to grow these small-diameter SWCNTs are present and active early in the growth, but not later. Previous studies, using both Raman spectroscopy^41^ and TEM imaging,^19^ have observed this behavior and have concluded that the absence of small-diameter SWCNTs at later times results from evolution of the size distribution of the catalyst particles toward larger sizes.

Another indication of catalyst particle evolution over time is a changing intensity ratio of the G-to-D bands of carbon. Figure 6e shows the ratio of the background-corrected intensities of the G and D bands, *I*_G_/*I*_D_, as a function of distance from the tops of the CNT mats. Previous work^41,42^ has shown that greater *I*_G_/*I*_D_ ratios in CNT mats are correlated with the presence of a larger fraction of SWCNTs, compared to multiwalled nanotubes (MWNTs), while lower *I*_G_/*I*_D_ ratios correlate with predominance of MWNTs. Figure 6e shows that the *I*_G_/*I*_D_ ratio of CNTs from Fe10 is in the range of 1–2 for measurements in the first 200 μm of growth and decreases to approximately 0.66 after 300 μm of growth. This observation indicates that SWCNTs present the first 200 μm of growth and their disappearance thereafter and provides further evidence that the size distribution of active Fe catalyst particles is changing over time. This distribution evolves from small sizes that produce SWCNTS to larger sizes that produce MWNTs over the course of CNT growth, losing small nanoparticles in the first 200 μm of growth so that only large particles continue growth thereafter. Note that previous work has found that, for CNT mats consisting of predominantly SWCNTs, the *I*_G_/*I*_D_ ratio observed in Raman spectra is normally much greater than the range observed here, typically in the range of 5–10 or higher.^42^ Our observation of the *I*_G_/*I*_D_ ratio in the lower range of 1–2 thus suggests that, even in regions of the mats that do contain SWCNTs, MWNTs are still the dominant form of CNTs present. This is consistent with the lack of SWCNTs observed in our TEM investigations.

By contrast, Raman spectra of CNT mats from Fe5W5 show no such evidence of catalyst particle size evolution. Figure 6 shows no peaks at any depth in the SWCNT breathing-mode region, indicating a lack of substantial numbers of SWCNTs throughout the mat structure. Furthermore, the *I*_G_/*I*_D_ ratio of the CNTs from Fe5W5 shows none of the time-dependent behavior seen for Fe10. Instead, the *I*_G_/*I*_D_ ratio remains constant at approximately 0.66, which is the value characteristic of pure MWNTs in the Fe10 sample. This suggests that the catalyst particles in the Fe/W catalyst do not undergo the size evolution from smaller to larger particles seen for pure Fe but rather maintain a more constant size distribution due to the stabilization against morphology evolution provided by the refractory stabilizer.

### AFM Analysis of Catalyst Particles

AFM imaging has been shown to provide valuable information on the morphology of particles from which CNTs nucleate and grow.^11,17,28,30,31,42,43^ We studied catalyst particle sizes and catalyst surface roughness and their evolution with time in the heated CVD environment for catalysts composed of both pure Fe and Fe combined with the refractory stabilizer. For these studies, catalyst-coated substrates were heated at 750 °C in a flowing Ar/H_2_ environment for different durations and then analyzed by AFM to assess particle sizes and surface morphology. Figure 7 shows AFM images of catalyst-coated substrates that have been heated in Ar/H_2_ for 45 min. The catalysts shown include Fe5W5 along with Fe10 (same total metal thickness) and Fe5 (same Fe thickness). Compared with the Fe5W5 sample, both Fe10 and Fe5 substrates appear visibly rougher. This suggests that more significant metal atom diffusion and surface roughening occurred in the absence of a stabilizer. We quantified these observations using root-mean-square (RMS) surface roughness measurements.^42^ The calculated RMS surface roughness values for these surfaces are as follows: Fe5W5, 1.05 nm; Fe10, 1.57 nm; Fe5, 1.68 nm.

**Figure 7:**
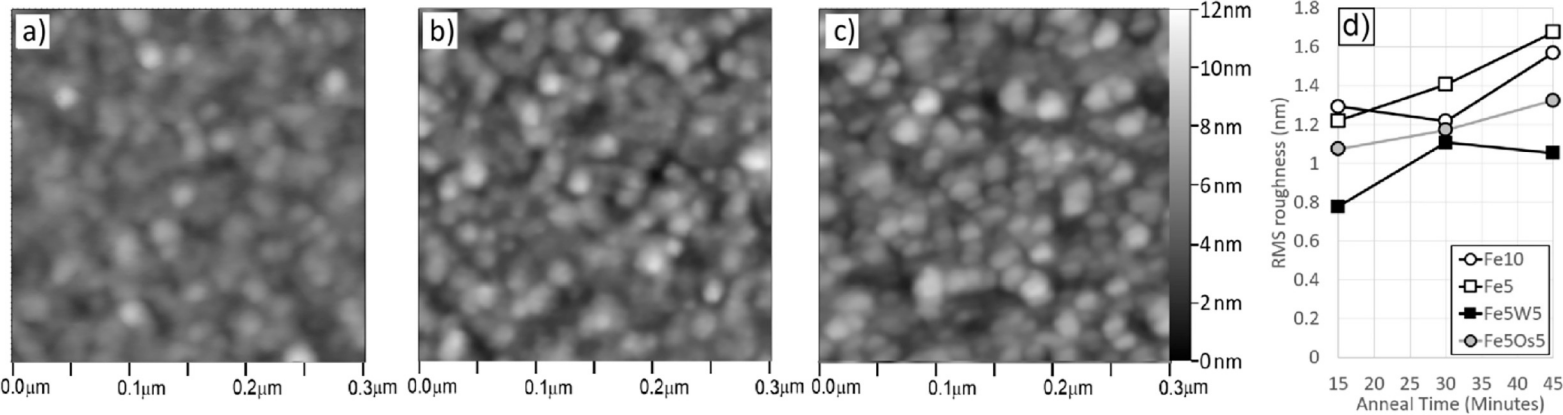
AFM images of catalyst-coated substrates heated in H_2_/Ar for 45 min. Vertical scale for al images is 0–12 nm height, as shown in panel (c). (a) Fe5W5, (b) Fe10, and (c) Fe5; (d) RMS surface roughness determined from AFM images for Fe10, Fe5, Fe5W5, and Fe5Os5 catalysts, after annealing at 750 °C in Ar/H_2_ for various times.

Also of interest is the time evolution of the surface roughness and the catalyst particle size range. Figure 7d shows the measured RMS surface roughness values determined from AFM images, for the catalysts Fe10, Fe5, Fe5W5, and Fe5Os5, after heating in Ar/H_2_ for periods from 15 to 45 min, which corresponds to the lower and upper limits of time spent at 750 °C in a reducing CVD environment in a typical CNT growth experiment. The data indicate that combining W with Fe results in lower surface roughness compared with pure Fe films. This is observed for films with either the same Fe thickness or the same overall metal thickness. More crucially, while all metal combinations show some level of increased surface roughness over time, the increase is significantly less for the Fe/W combination. This suggests that tungsten effectively stabilizes the morphology and size of the Fe particles over time. The Fe5Os5 catalyst exhibits similar behavior, showing both lower initial surface roughness and a reduced rate of roughness increase over time when compared to that of pure Fe films. This confirms osmium’s effectiveness as a stabilizer for iron.

It should be noted again that, while these studies do show that W and Os stabilize the Fe catalyst against morphology and particle-size changes, one must be cautious of drawing *quantitative* conclusions about the behavior of these films in the actual CVD environment of CNT growth. The inclusion of carbon in the gaseous feedstock and the subsequent interactions of C atoms with the metal films can also substantially affect catalyst particle size distributions and surface roughening dynamics.^9^ Nevertheless, the above results show that inclusion of a refractory stabilizer can significantly slow changes to catalyst particles and hence prolong their active lifetime for CNT growth.

## Discussion

Our AFM results demonstrate that addition of high-melting-point refractory metals stabilizes nanometer-scale particles of catalytic metals like iron, allowing them to maintain a constant size for longer periods of time in a high-temperature reducing environment such as is used in CNT growth. Our Raman results confirm that this size stabilization also stabilizes the CNT growth chemistry, especially with respect to changes in the size distribution of the particles, resulting in a distribution of CNT diameters that is more stable in time. Our studies show that nanoparticles of Fe combined with refractory metals are more stable in size and chemistry. This stability results in longer growth times and ultimately longer CNTs. We also see that the mixed metal catalysts (Fe + refractories) tend to be less catalytically active than pure Fe, resulting in a slower CNT growth rate. Whether longer CNTs are achieved with a given mixed-metal combination therefore depends on the trade-off between increased growth time and decreased growth rate. In the case of Fe/W mixed-metal catalyst, we do indeed achieve longer CNTs than are given by a pure Fe catalyst alone.

This group’s previous work used refractory-stabilized catalyst to achieve longer CNTs, with CO used as the carbon feedstock gas.^37^ In that work, it was postulated that under our conditions, the primary mechanism for catalyst particle deactivation was Ostwald ripening of the catalyst particles until they were either too small or too large to support CNT growth. The refractory was postulated to stabilize the catalyst by acting as a “diffusion inhibitor” (or, more generally, an “Ostwald Ripening inhibitor”): the refractories cause the catalyst metal atoms to bind more tightly to the particle, making these atoms less likely to detach, diffuse away across the surface, and bind to another potentially larger particle, i.e., less likely to do Ostwald ripening. Slowing the Ostwald ripening allows catalyst particles to remain active for a longer period. This longer activity results in a longer final length for the CNTs, provided that the increase in particle lifetime compensates for the typically slower growth rate observed in mixed-metal catalysts. This mechanism is illustrated schematically in Figure 8.

**Figure 8:**
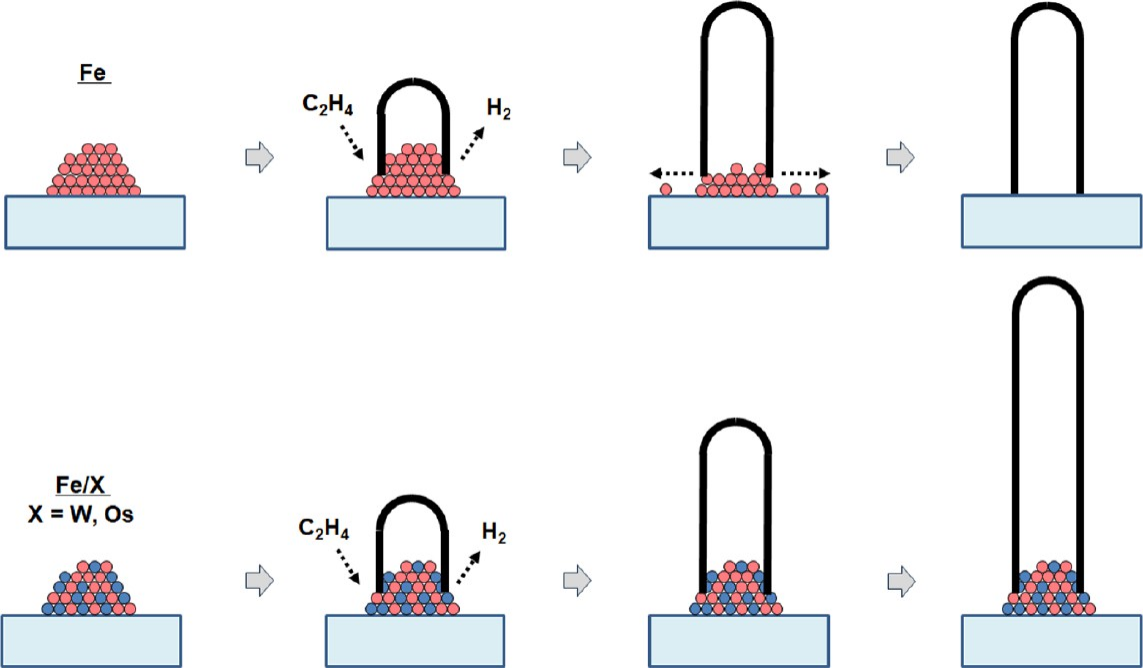
Schematic Illustration of the mechanism for longer CNT growth resulting from catalyst particle stabilization by heavy refractory diffusion inhibitor metals. The heavy diffusion inhibitor atoms bind and hold the catalyst atoms in place, preventing catalyst particle erosion by diffusion of the catalyst atoms away from the particle.

All of our current results, achieved using C_2_H_4_ as carbon feedstock, agree with this “Ostwald ripening inhibitor” hypothesis. Our AFM results show that addition of refractory to Fe slows the coarsening of the catalyst, as expected if the refractory is slowing Ostwald ripening. This effect would allow the particles to remain for longer times within the size range appropriate for CNT growth, hence the increased CNT growth time. Inhibition of Ostwald ripening also correlates with our observations in CNT alignment and Raman spectra. Fe/W particles are stable for a much longer time than Fe particles alone, affecting both CNT alignment and catalyst particle size evolution.

Observation of exclusively Fe in nanoparticles incorporated into CNTs grown from Fe/W shows that W is indeed resistant to diffusion or any morphological change that would result in mixed-metal particles detaching from the substrate to incorporate into CNTs. The fact that strictly Fe is observed in the CNTs grown from catalyst that starts as an intimate atomic-level intermixing of the Fe and W atoms (based on the method of catalyst preparation used) suggests that Fe incorporation into the CNTs may arise primarily from the diffusion of individual Fe atoms into the CNTs during growth (to agglomerate into particles inside the CNTs as growth proceeds) rather than detachment of whole catalyst particles from the surface. This is consistent with our notion of W as a catalyst stabilizer and diffusion inhibitor. An alternative explanation could be proposed in which the catalyst particles are composed exclusively of iron with W on the surface simply acting as an additional support matrix, in combination with or in addition to the alumina, to keep the Fe from diffusing and aggregating. However, this appears unlikely given atomic-level intermixing of the metal atoms in the catalyst preparation method and the existence of several stable Fe/W intermetallic compounds in this temperature and composition range (see below). We did attempt to image directly the particles giving rise to CNT growth by ripping the CNTs away from the substrate using tweezers and imaging the ends with TEM; however, all that was seen in these attempts was open-ended CNTs at the base end of the growth, with no entrained particles, implying better adhesion between particles and substrate than between particles and CNTs.

Also important to maximization of CNT length is optimization of the temperature for CNT growth. Our previous work^38^ showed 750 °C to be the optimum temperature for growth of CNTs from pure iron in our CVD system; however, CNT ultimate length vs temperature has not yet been optimized for any of the mixed-metal catalysts. Temperature-dependent growth measurements combined with kinetic modeling^44^ have suggested that, for any catalyst, ultimate CNT length vs temperature will follow a “volcano plot,” with terminal CNT length rising to a maximum, then falling off, as temperature is increased. The higher-stability mixed-metal catalysts may yield a maximum CNT length at higher temperature compared to pure Fe, so that the ultimate CNT lengths for CNTs reported here may not be the global maximum length accessible with respect to temperature for these catalysts. Future experiments will probe the temperature dependence of CNT growth using these catalysts.

The details of the mechanism of stabilization by the refractory will, of course, depend on the nature of the interactions between the catalyst atoms and the refractory atoms. For example, the Fe/W phase diagram^48^ shows that, for Fe:W atom ratios near 1:1 at these CVD temperatures, the mixture exists in the form of the intermetallic compound Fe_7_W_6_, with any excess Fe or W present in the form of Fe_2_W or pure W, respectively. Fe_7_W_6_ is stable as a solid up to approximately 1700 °C, substantially higher than the melting point of pure Fe (1538 °C). This indicates that the Fe atoms are bound more tightly within the lattice of Fe_7_W_6_ than they are in pure Fe, stabilizing the Fe/W particles against morphological changes at CNT growth temperatures. The operative morphology changes that end CNT growth prematurely under our conditions may involve Fe atom diffusion and Ostwald ripening but could also involve mechanisms such as migration of the particles into the substrate surface or merging of catalyst particles. Ongoing studies will aim to further elucidate the details of the catalyst stabilization mechanism.

Finally, we note that some potential refractory stabilizers, most notably osmium, have a high cost (>$100/gram), which may prohibit their adaptation into any large-scale process to manufacture CNTs. Tungsten, on the other hand, is commercially available for less than $0.20/gram. Costs of catalysts will certainly be a consideration in efforts to scale up CNT production to industrially relevant levels and may influence choice of refractories used in ongoing research to scale up processes such as described in this work.

## Conclusions

Growth of dense mats of CNTs by catalytic CVD has been investigated using catalyst films composed of mixtures of catalytically active iron combined with the refractory metals W and Os, to investigate inclusion of refractories to stabilize the catalyst particles from which CNTs grow and thus to grow longer CNTs. It was found that both W and Os extend the lifetime of the catalyst particles but that both also result in a slower CNT growth rate. Whether the resulting CNTs are longer depends on the balance between the increased catalyst particle lifetime and decreased CNT growth rate. In the case of tungsten, the decrease in CNT growth rate is more than offset by the increase in growth time, resulting in a greater ultimate length of the CNTs grown. In the case of osmium, the ultimate CNT length achieved is approximately the same as that given by CNTs grown from pure Fe, suggesting that these two competing effects largely cancel each other in this case. It was also found that even very thin layers of Fe catalyst, too thin to grow CNTs on their own, would grow CNTs to substantial lengths if combined with W or Os, again showing the stabilizing effect of the refractory on the particles of the catalyst. This effect was observed for mixed-metal catalysts consisting of as much as 85% refractory and only 15% Fe catalyst.

Position-dependent micro-Raman spectroscopy showed that the catalyst particles of pure Fe giving rise to CNT growth were evolving and growing larger over the lifetime of the CNT growth, whereas catalysts of Fe combined with W showed no such time evolution but were relatively stable over the growth lifetime. Greater stability of the Fe/W particles compared to pure Fe was also shown by position-dependent SEM imaging, which showed a much slower onset for the Fe/W catalyst of a loss of CNT alignment in the growing CNT films. TEM imaging showed that the CNTs grown were primarily double-walled CNTs, for both the pure Fe catalyst and mixed-metal catalysts. AFM measurements of catalyst films subjected to CNT growth conditions (high-temperature reducing environment) without the addition of C_2_H_4_ showed that films of Fe combined with refractory evolved and roughened much more slowly than films of pure Fe, showing the stabilizing effect of refractory with respect to Fe atom diffusion and Ostwald ripening of the catalyst particles.

These results show that refractory stabilizers can increase the lifetime of catalyst particles in metal-catalyzed CNT growth and can result in the CNTs reaching greater ultimate lengths. We suggest a general model for this behavior, in which the refractories inhibit catalyst-atom surface diffusion, catalyst particle Ostwald ripening, and other particle morphology changes that would otherwise result in deactivation of the particles. Research of these effects is ongoing, with the ultimate goal of producing dense mats of CNTs with lengths of many tens of centimeters to meters, sufficient for the applications envisioned for these materials.

## Materials and Methods

### Catalyst/Substrate Preparation

CNTs were grown on substrates consisting of silicon wafers coated with 150–200 Å (15–20 nm) of aluminum oxide and 1–10 Å (0.1–1.0 nm) of catalyst metal consisting of either pure iron or iron mixed with tungsten or osmium in controlled proportions. Note that, in general, a barrier layer of Al_2_O_3_ or some other refractory material is desirable or necessary to grow CNTs on Si or SiO_2_ substrates, to prevent excessive diffusion of the catalyst into the substrate.^11^ The substrates were 500 μm-thick prime-grade Si wafers topped with 1 μm of wet thermal SiO_2_ (University Wafer, Inc.). Aluminum metal was evaporated onto these wafers using an electron-beam evaporator (Varian) and then oxidized to Al_2_O_3_. Al was evaporated in layers of 3–5 nm, with the metal evaporator system vented to room air for 10 min between layers to allow the thin Al of each layer to oxidize. Typical complete Al_2_O_3_ films consisted of 3 such layers, for a total Al film thickness of 9–15 nm, for which complete oxidation to Al_2_O_3_ was ensured by air exposure between layer depositions. The Al_2_O_3_-covered wafers were cut into square samples of size 1.6 cm × 1.6 cm, which served as the substrates for metal catalyst deposition and CNT growth.

Catalyst and refractory metals were spun onto these square substrates from toluene solutions of organometallic compounds containing the metal atoms of interest. Spin-on of these solutions was done with a benchtop spin-coater system (KW-4A, ChemMat Technology Inc.). The solutions were made by codissolving the organometallic compounds with polystyrene binder polymer. The organometallic compounds used were the following: for Fe, Iron­(III) acetylacetonate, Fe­(C_5_H_7_O_2_)_3_ ; for W, tungsten hexacarbonyl, W­(CO)_6_ ; for Os, dicyclopentadienyl osmium (osmocene, (C_5_H_5_)_2_Os) or osmium carbonyl ((CO)_12_Os_3_). Polystyrene (*M*_w_ = 35000, Aldrich Chemicals) was used as the binder polymer to ensure smooth, uniform coating of the spun-on films. A typical spin-on solution would contain, for example, 5–10 mM Fe­(C_5_H_7_O_2_)_3_, 5–10 mM W­(CO)_6_, and 30 g/L polystyrene, all dissolved in toluene solvent. These solutions gave uniform polymer/organometallic film thickness of 0.120 μm when spun on at 4000 rpm. Given the known concentration of organometallic compounds and polymer in the solution, as well as the thickness of the resulting film, and assuming the density ratio of organometallic to polymer molecules in the spun-on film mirrors that in the solution, we could readily calculate the areal density of metal atoms on the substrate. This, in turn, allowed us to determine the thickness of the metal film once the organic material was removed.

After the catalyst solutions were spun onto the square wafer substrates, the substrates were immediately transferred to a plasma etching system (PE25-JW, Plasma Etch, Inc.) and exposed to an O_2_ RF plasma, which etched away the polymer and organometallics’ ligands to leave behind thin films of oxides of the metals in the solutions. These metal oxide films were very stable and could be stored for many months without degradation in performance. Upon exposure to a reducing environment at elevated temperatures in the CVD-growth apparatus, these metal oxides were reduced to thin films of the elemental metals of thickness predetermined, as discussed above, by the concentrations in the spin-on solutions. For CNT growth experiments, the square wafer substrates were cleaved into smaller samples, typically 2–3 mm on a side. These small samples were used in individual CNT growth experiments: their small size allowed up to 12 samples of different catalyst compositions to be processed in a single CNT growth experiment.

### CNT Growth

CNTs were grown on these substrates in a CVD reactor. This reactor comprised a 1 in. diameter, 24 in. long thick-walled quartz tube fitted with 1 in. Ultra-Torr end flanges (Swagelok Corp.) and passed through a 1 in. diameter split-hinged tube furnace (MiniMite, Thermo Scientific). Gases including Ar, H_2_, and C_2_H_4_ were flowed through the quartz tube via a system of valves and fittings, with flow controlled and measured by mass flow controllers (GE50 series controllers with Series 946 Vacuum System Controller, MKS Instruments, Inc.). Pressure during CNT growth was maintained at 1 atm by simply directing the exhaust gas flow to vent into a building system vent open to the atmosphere.

For CNT growth, the substrates were placed onto a quartz slide, which was then loaded into the center of the furnace’s hot zone, inside the quartz tube. CNTs were grown by passing mixtures of C_2_H_4_, hydrogen, and argon through the quartz tube and over the substrates at elevated temperature, typically 750 °C, at a pressure of 1 Atm. Under these conditions, the thin layers of metal form nanoparticles with diameters of a few nanometers, upon which the ethylene decomposes to release its carbon atoms, which form into CNTs. This process results in dense mats of CNTs, which nucleate and grow parallel to each other as they grow perpendicularly away from the substrate surface.

CNT growth runs were carried out as follows. After substrate samples were loaded into the quartz tube and the system was evacuated by a mechanical pump, the system was filled with flowing Ar (100 sccm) and H_2_ (25 sccm) to 1 atm pressure and then flushed with these gas flows for 25–30 min. The tube furnace was then turned on, and reached the CNT growth temperature of 750 °C in 11 – 12 min. At this temperature in a hydrogen-containing atmosphere, the metal oxides will quickly reduce to thin films of elemental metals, which then form into nanometer-sized particles. These nanoparticles of catalytic metal then serve as nucleation centers for the CNTs. The samples were allowed to anneal in an Ar/H_2_ flow for 15 min at 750 °C to ensure completion of the metal reduction and catalyst particle formation and stabilization process. At this point, C_2_H_4_ (25 sccm) was added to the Ar/H_2_ gas flow, commencing the CNT growth, for 2–60 min. The C_2_H_4_ and H_2_ flows were then shut off, the Ar flow was increased to 150–300 sccm to flush the system, the tube furnace was switched off, the system was cooled, and the CNT samples were retrieved for analysis.

### Characterization of CNTs

The CNTs were characterized using scanning electron microscopy (SEM), transmission electron microscopy (TEM) with energy-dispersive X-ray spectroscopy (EDS), and micro-Raman spectroscopy. SEM (Model 6010LA, JEOL Ltd.) was used to measure the lengths of the CNT in the CNT mats and at high resolution to characterize the detailed morphology of the CNTs within the mats. The TEM systems used were a 300 kV aberration-corrected S/TEM (FEI Titan) and a 200 kV JEOL NEOARM STEM for detailed CNT characterization and for counting the number of walls in the CNT, as well as for elemental analysis of metal particles that were occasionally found entrained in the CNTs after growth. CNTs were prepared for TEM analysis by sonicating samples of the CNT mats in isopropyl alcohol until the CNTs were dispersed and then drop-casting the suspended CNTs onto TEM grids (3 mm Cu grids, 200 μm pitch, lacey carbon coated: TED Pella, Inc.).

Raman spectra were measured by using a custom-built micro-Raman setup. Samples were excited with a continuous wave diode-pumped solid-state laser (Excelsior, Spectra Physics, 532 nm, 100 mW) through an upright microscope using a 100× objective with a numeric aperture of 0.9. The laser power incident on a sample was maintained at ∼100 μW to reduce the possibility of heating and damaging the samples while acquiring spectra. The scattered Raman light was analyzed using a spectrometer (Spectra Pro 2300i, Acton, *f* = 0.3 m) coupled to the microscope and equipped with a grating (1800 grooves/mm) and a CCD camera (Pixis 256BR, Princeton Instruments).

### Characterization of Catalyst Particles

In separate experiments, catalyst-coated substrates were exposed to conditions identical to those used to grow CNTs, except that ethylene was not used: substrates were exposed to flowing Ar/H_2_ at 750 °C in the CVD apparatus for times comparable to those used for CNT growth, but without introduction of C_2_H_4_ to cause CNT growth. Catalyst particles will still form and evolve in this heated, reducing environment, analogous to the process occurring in CVD growth, but the catalyst particles are produced without attached CNTs, leaving them amenable to AFM analysis. Catalyst stabilization and Ostwald ripening inhibition will still result in predictable differences in the size distributions of the catalyst particles, as when CNTs are grown. Note that, because carbon can also affect diffusion of metal atoms, quantitative comparison between catalysts processed with vs without ethylene is not possible; however, qualitative differences and trends in catalyst particle behavior are still discernible. These catalyst particles were characterized by using atomic force microscopy (AFM, Cypher AFM, Asylum Research and Oxford Instruments Company). We used AC240 cantilevers with a nominal spring constant of ∼2 N/m and free resonance frequency of ∼70 kHz to determine catalyst particle sizes and surface roughness.

## Supplementary Material



## Notes

CNT growth curves (CNT length vs time) for catalysts composed of Fe/Os, and TEM and elemental analysis of CNTs and metal particles (PDF)
