# Supplementary material for: Growth of Carbon Nanotubes Using Mixed-Metal Catalysts That Include Heavy Refractory Metals as Catalyst Stabilizers
Source: ACS Omega. 2025 Sep 2;10(36):40968–80. doi: 10.1021/acsomega.5c03084 (PMC12444553; doi:10.1021/acsomega.5c03084)
Supplement: Supplementary file 1 [file ao5c03084_si_001.pdf]

# Growth of carbon nanotubes using mixed-metal catalysts that include heavy refractory metals as catalyst stabilizers

Wonjung Park[1], Matthew G. Boebinger[2], Liam Collins[2], Alexander A. Puretzky[2], Ilia N. Ivanov[2], David B. Geohegan[2] and Michael J. Bronikowski[1]*

[1] Dept. of Chemistry and Biochemistry, University of Tampa, Tampa FL 33543

[2] Center for Nanophase Materials Science, Oak Ridge National Laboratory, Oak Ridge, TN 37830

* Corresponding author. *Email address*: mbronikowski@ut.edu

## **Supporting Information**

**Supporting Information**

*S1. CNT length vs time for Os catalyst stabilizer*

Figure S1 shows measured CNT length vs growth time for the Fe5Os5 mixed-metal catalyst. For ease of comparison, results for the Fe5 and Fe10 catalysts [40] are reproduced here as well.

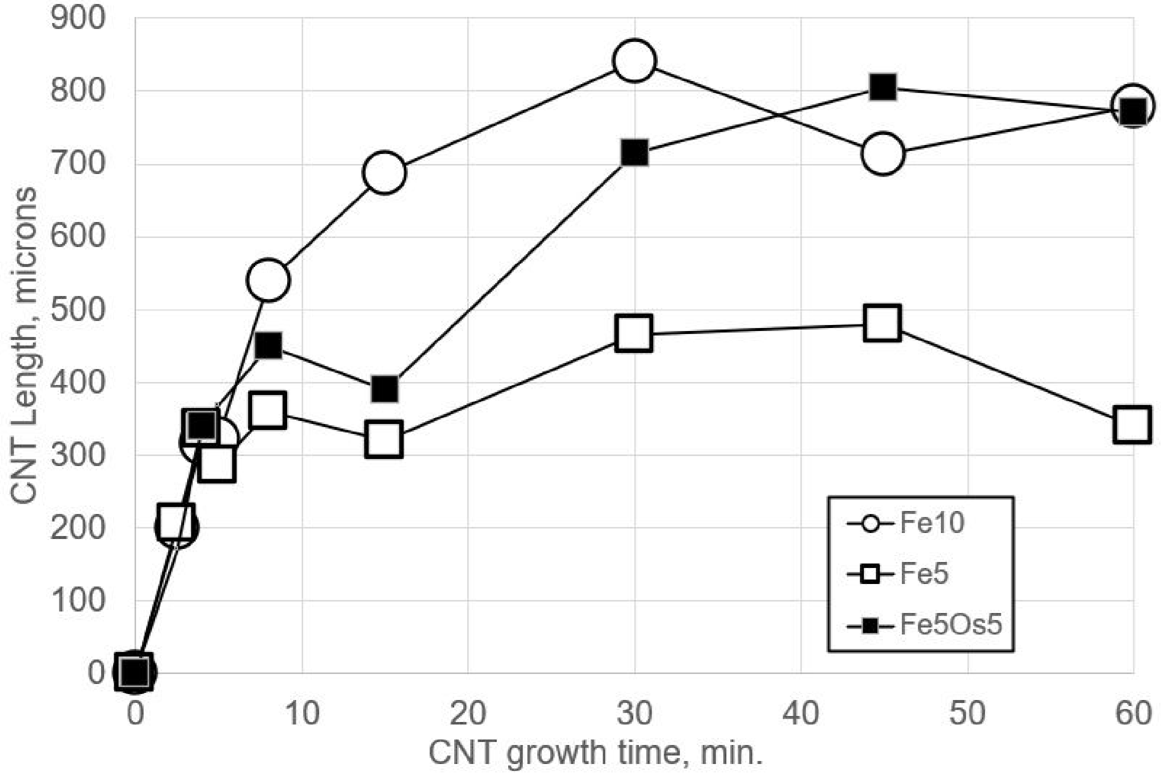


**Figure S1.** CNT length vs growth time for catalysts of pure iron and Fe/Os mixed metal. Plots for Fe10 and Fe5 are reproduced from reference 40 with permission.

Results for the Fe5Os5 catalyst show effects like those seen for Fe5W5, though decidedly less pronounced. CNTs from Fe5Os5 appear to continue lengthening for approximately 30 min., and reach an ultimate length of approximately 750 microns, both substantially greater than 4 min. of growth and 400 microns final length observed for 5 Å Fe alone. However, comparing Fe5Os5 to Fe10, while the CNT growth time appears greater, the ultimate length reached is somewhat less than that achieved by the Fe10 film. There are two potential explanations for this result. One possibility is that osmium is simply a less effective stabilizer for iron catalyst compared to tungsten. The second

possibility is that the presence of osmium may decrease the overall catalytic activity of the iron more than tungsten does. Nevertheless, the fact that the combination of 5 Å of iron with 5 Å of osmium yields a longer growth time and CNT length than 5 Å of iron alone indicates that osmium does indeed contribute some catalyst stabilization, allowing catalyst particles to remain active longer and facilitating growth of longer CNTs than what is achievable in its absence.

*S2. TEM and Elemental analysis of metal particles incorporated into CNTs*

Occasional metal nanoparticles were observed incorporated into the CNTs in all samples analysed by TEM. To investigate these metal particles, samples of CNT mats grown from Fe5W5 catalyst were imaged on TEM grids without dispersion by sonication, so that CNTs within the mats could be studied directly at different well-defined times in their growth by imaging at various points along their lengths. For these analyses, thin sections of mats of CNTs grown to the full length achieved by Fe5W5 catalyst (approximately 1 mm) were remove from CNT mats using tweezers and laid flat on TEM grids. It was found that, to assure good adhesion of the CNT sample to the TEM grid, it was necessary to cover the CNTs with a second TEM grid (the "cover grid"), press down, then briefly sonicate the grid/CNT/grid "sandwich" assembly in isopropyl alcohol before removing the cover grid. These CNT samples would then remain stable and unmoving during imaging by TEM.

Position-dependent imaging of these CNT mat samples and their entrained metal particles showed essentially no particles at the tips of CNTs (farthest from the substrate surface), with a slow increase in the number density of the particles as the CNTs were imaged farther from the tips. Within 100 – 200 microns of the base of the CNTs (closest to the substrate surface) particles were quite common. Hi-resolution TEM imaging of the particles revealed lattice fringes whose spacings were consistent with inter-atomic layer spacings in metallic iron. Figure S2 shows an example of a particle imaged in this way. This figure also shows an image of the same particle acquired in scanning-TEM high angle annular dark field (STEM-HAADF) mode. Here, image brightness denotes intensity of scattered electrons collected by an annular detector, with greater brightness generally denoting a heavier element.

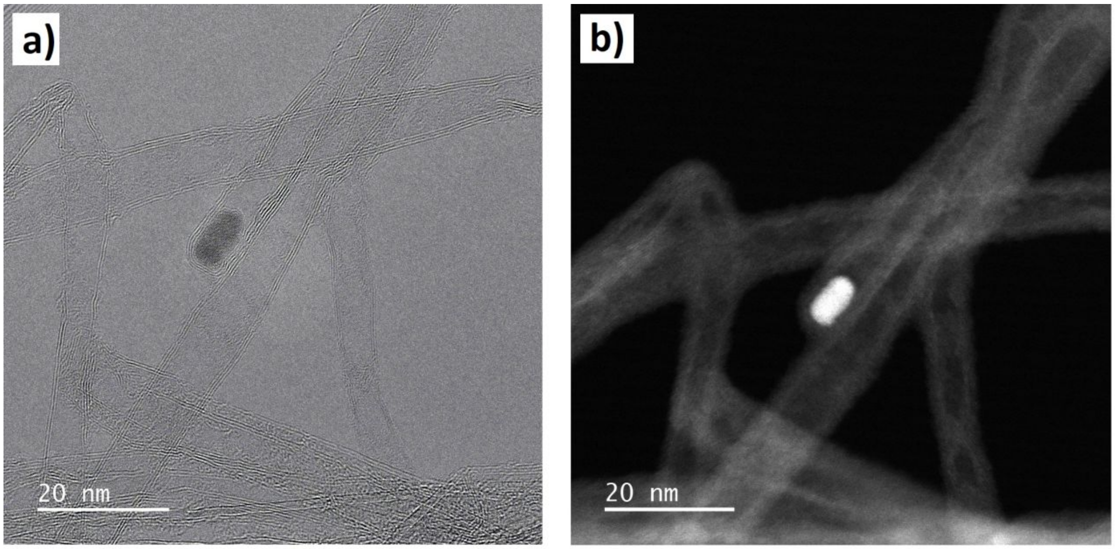


**Figure S2.** Nanoparticle entrained in a CNT grown from Fe5W5 catalyst.
a) TEM image    b) STEM-HAADF image

Elemental analysis of these samples was carried out with EDS using a large-angle SDD detector while operating at 200kV with a current of approximately 90pA. The samples were imaged in STEM-HAADF mode (not in transmission mode) while the EDS detector was engaged. STEM-HAADF imaging (Figure S2) was used due to the higher spatial resolution for detecting EDS signal at precise locations, and it additionally has the advantage of producing very high contrast between metal particles and the carbon nanotubes, making the particles easy to identify. Figure S3 shows a STEM-HAADF image of a region near the base of the Fe5W5 CNT mat, with metal particles clearly visibly as bright spots. Also shown in Figure S3 is the EDS spectrum acquired as the scanning electron beam was scanned over this area for approximately 300 sec. Aside from C and O K-a lines, substantial signals are seen for Si, Fe and Cu K-lines, Fe and Cu L-lines, and W M-lines, indicating that all these elements are present in the sample. As no Cu was used in the catalyst preparation or CNT growth, the most likely source of Cu in the samples is the copper TEM grids, which may have shed small amounts of Cu during the sonication. Si in the sample likely arises from the substrate, small particles of which may have been removed along with the CNT mat sections. This observation suggests that some fraction of the Fe and W signal may arise from residual catalyst on the substrate surface, which remains after growth and may have been removed along with the Si when the CNT mats were removed.

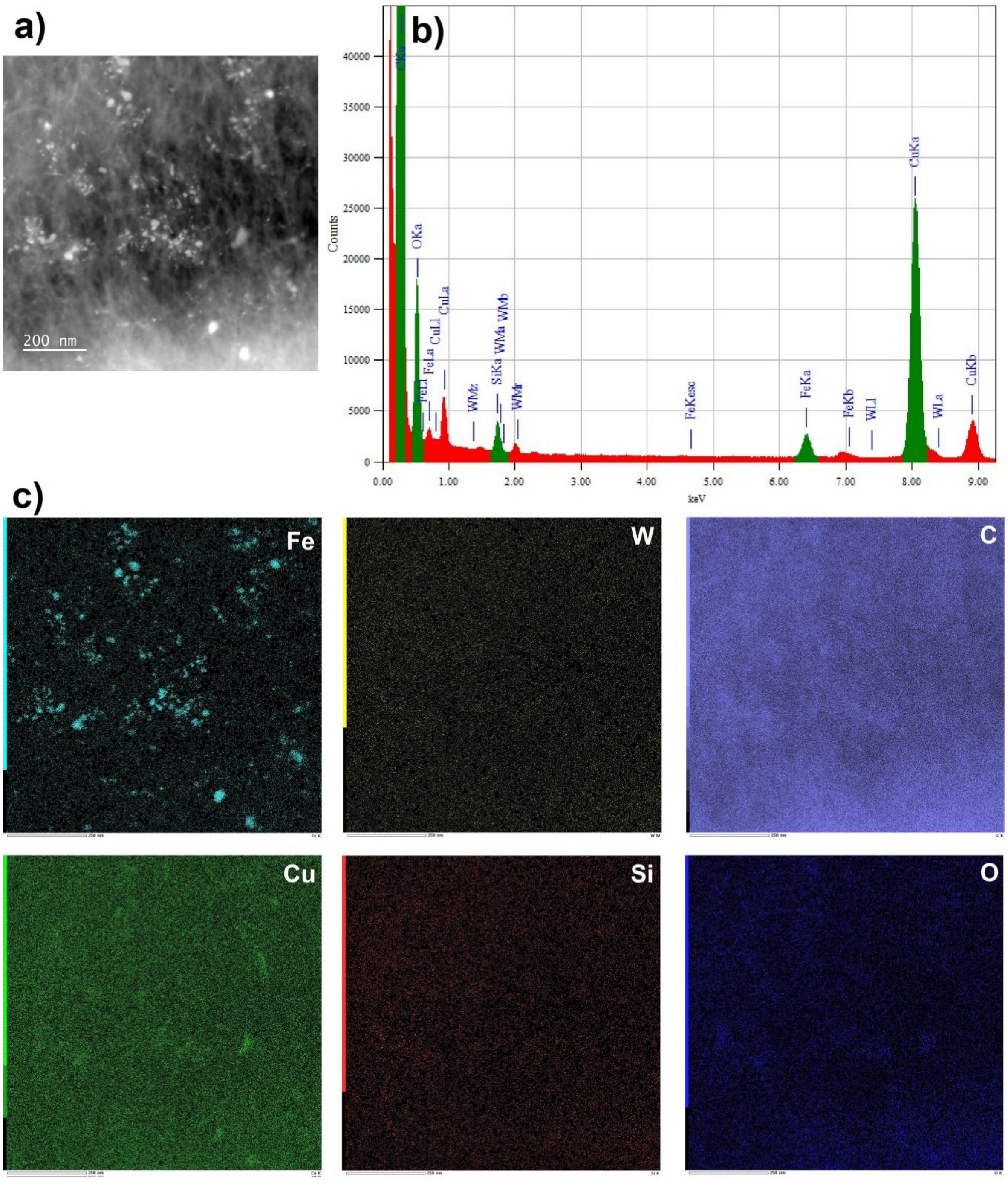


**Figure S3.** a) STEM-HAADF image of CNT mat grown from Fe5W5, acquired near base of CNTs. Metal particles are visible as bright spots.

b) EDS Spectrum acquired while scanning the electron beam over the area shown in image a)

c) Elemental maps from CNT mat grown from Fe5W5. Region mapped is the same as in image a), and scalebar of a) also applies to images in c)

Mapping the EDS signal from peaks of various elements as a function of position of the scanning beam yields maps of the relative abundance of each element on the scanned surface of the sample. Figure S3 also shows such elemental maps for this same region for Fe and W, as well as C, Cu, Si and O. These maps show that most of the particles consist exclusively of Fe, with none giving any signal above background for the W line. The map for Cu also shows that a few of the particles visible in image S3 consist of Cu, which again probably arise during the processing to prepare these grids. The C signal map shows the CNTs of the image, while Si and O show only a non-specific low-level background signal that presumably arises due to traces of these elements (in the form of $SiO_2$ from the substrate) entrained in the CNT mat when it was harvested. The W map also shows such a low-level background signal, with no bright spots corresponding to particle positions, which again suggests that traces of W (along with Fe) may have been entrained in the sample during harvesting.

This same pattern was observed for particles imaged anywhere along the length of the CNTs in the mat. Figure S4 shows a STEM-HAADF image of CNTs with entrained particles that was acquired near the middle of the CNTs' length, approximately 500 microns from the base of the CNTs. The particles are much sparser in this region, but two can clearly be seen. Figure S4 also includes elemental maps for Fe, W and C for this region. Again, the particles only show signal for Fe, with no spots in the W map at the particle locations. This image was acquired at sufficiently high magnification that the CNTs themselves can be distinguished in the C elemental map. All such images at all points investigated in the Fe/W mat showed this same pattern, demonstrating that the particles entrained in the CNTs during growth were composed exclusively of Fe.

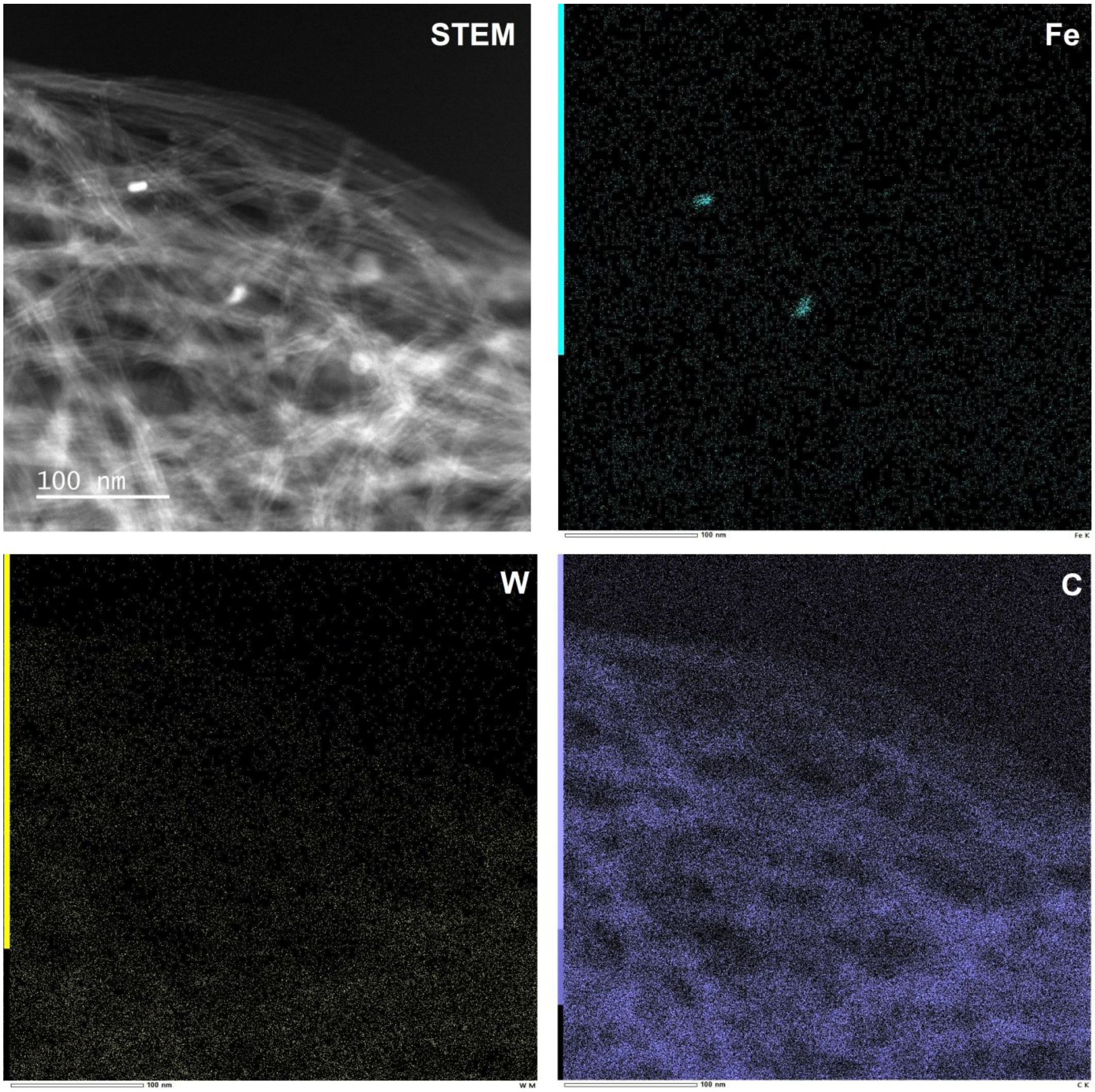


**Figure S4.** STEM-HAADF and elemental maps from CNT mat grown from Fe5W5, at a location approximately 500 microns from CNT bases (half-way from bases to tips). Elemental maps show same as STEM image, and scalebar in STEM image also applies to elemental maps.
